# Clinical features and management of nonfunctioning giant pituitary adenomas causing hydrocephalus

**DOI:** 10.18632/oncotarget.24171

**Published:** 2018-01-11

**Authors:** Danfeng Zhang, Jigang Chen, Zhenxing Li, Junyu Wang, Kaiwei Han, Lijun Hou

**Affiliations:** ^1^ Department of Neurosurgery, Changzheng Hospital, Second Military Medical University, Shanghai, 200003, China

**Keywords:** nonfunctioning giant pituitary adenomas, hydrocephalus, clinical features, management, surgical approach

## Abstract

We evaluated the features of clinically nonfunctioning giant pituitary adenomas (NFGPAs) causing hydrocephalus to highlight the timing of hydrocephalus management and surgical approaches. A total of 24 patients with NFGPAs and hydrocephalus were included. Eighteen patients underwent endoscopic transsphenoidal surgery. Ten patients received pterional surgery, including 6 patients as first treatment and 4 cases with recurrence after transsphenoidal approach. Gross total resection was achieved in 10 patients, including 6 cases (6/18, 33.3%) with endoscopic transsphenoidal surgery and 4 cases (4/10, 40%) with pterional surgery. All patients were divided into preoperative EVD group and non-preopoerative EVD group. The proportion of patients receiving postoperative EVD or shunt was significantly higher in non-preoperative EVD group than that in preoperative EVD group (9/15 vs. 1/9, *P* = 0.033). Visual impairment score (VIS) was evaluated for each patient. We detected significant vision improvement according to the preoperative and postoperative VIS (median, interquartile range: 62, 48.25–77 vs. 36.5, 0–50.75, *P* < 0.001). Conclusively, for patients with NFGPAs and hydrocephalus, preoperative EVD might reduce the need for a second shunt or EVD. Surgical approach should be decided based on the clinicoradiological features and surgeons’ experience for individualized treatment, and endoscopic transsphenoidal resection of pituitary adenomas was suggested for most NFGPAs.

## INTRODUCTION

Clinical case-finding studies have suggested that the prevalence of pituitary adenomas ranges from 1/865 to 1/2688, among which 15% to 54% pituitary adenomas are clinically nonfunctioning [1–7]. Generally, nonfunctioning pituitary adenomas (NFPAs) are asymptomatic at early stage, thus, at the time of diagnosis, most patients with NFPAs present with significant headache, visual disturbance and hypopituitary due to the large size of the tumors [2]. NFPAs with the diameter exceeding 40 millimeter (mm) are considered as giant, which are rare and frequently invade suprasellar structures, leading to symptoms of mass effect [2, 8].

Nonfunctioning giant pituitary adenomas (NFGPAs) causing hydrocephalus are even rarer. A detailed search of Medline using terms “hydrocephalus” and “giant pituitary tumor OR giant pituitary adenoma OR giant hypohysoma” produces only several case reports, with inconsistent conclusions regarding the timing of hydrocephalus management and surgical approaches [9–13]. It was assumed by Laws that pituitary adenomas were usually soft and would descend to intrasellar region, unblocking the third ventricle and rendering presurgical external ventricular drainage (EVD) unnecessary [9]. Contrastly, Joshi et al and Verhelst et al considered presurgical EVD to be necessary and safe for subsequent surgery [9, 11]. Most reported cases were operated by the transsphenoidal approach with a low morbidity and mortality, while tumors with dumb-bell-shaped or extensive suprasellar invasions were difficult to resect with endonasal approach and surgeons might turn to transcranial or combined approaches [9, 12]. Endoscopy and neuronavigation system have also been described to be helpful in the resection of NFGPAs [8, 12].

In the present study, we evaluate the management of hydrocephalus, clinical features and surgical approaches of NFGPAs causing hydrocephalus in order to highlight the proper treatment of this rare condition.

## RESULTS

### Demographics and clinical features of NFGPAs complicated with hydrocephalus

Fourteen males (58.3%) and 10 females (41.7%) with a mean age of 53.75 years (range: 25 to 69 years) were included in our study (Supplementary Table 1). According to our results, tumor mass were found to be positively associated with the vision improvement (Rs = 0.597, *P* = 0.002), while presurgical hypertension (*P* = 0.480), diabetes (*P* = 0.722), hyperlipidemia (*P* = 0.393), alcohol (*P* = 0.216), smoking (*P* = 0.750), tumor extension (*P* = 0.799) were not the related factors. We detected no statistical difference in terms of vision improvement for patients treated by 3 different neurosurgeons (*P* = 0.504) (Table 1).

**Table 1:** Comparison of vision improvement in different groups

	Hypertension		Diabetes		Hyperlipidemia		Alcohol		Smoking		Tumor extension		Operators		
	Yes	No	Yes	No	Yes	No	Yes	No	Yes	NO	Yes	No	1	2	3
Number of patients	8	16	5	19	4	20	6	18	7	17	7	17	7	10	7
Vision improvement (median, IQR)	34, 23.25-45.75	32, 20-41	34, 24-44	34, 9-45	34, 24.25-43.75	25, 18.5-41.25	28, 19.5-44.25	39, 33-43.75	34, 23.5-44	30, 20-44	34, 20.5-44	34, 24-44	30, 26-43	39, 18-47	25, 20-43
*P* value	0.480		0.722		0.393		0.216		0.750		0.799		0.504		

Abbreviations: VIS, visual impairment score; IQR, interquartile range

Gross total resection (GTR) was available in 6 cases (6/18, 33.3%) among patients receiving endoscopic transsphenoidal surgery and 4 cases (4/10, 40%) among patients undergoing pterional surgery, respectively. All patients were divided into preoperative EVD group and non-preoperative EVD group. One case (1/9, 22.2%) received postoperative ventriculoperitoneal shunt (VP shunt) due to sustained hydrocephalus in preoperative EVD group. Contrastly, in non-preoperative EVD group, 9 cases (9/15, 60%) underwent postoperative EVD or VP shunt due to acute or aggravated obstructive hydrocephalus. The proportion was significantly higher in non-preoperative EVD group than that in preoperative EVD group (*P* = 0.033).

All patients presented with visual defect on admission. According to the preoperative and postoperative visual impairment score (VIS), we detected significant vision improvement (median, interquartile range: 62, 48.25-77 vs. 36.5, 0-50.75, *P* < 0.001). Recovery of visual disturbance was achieved in 22 patients (91.7%), among whom 7 obtained complete recovery (31.8%, postoperative VIS: 0) and 15 had partial recovery (68.2%). Two patients obtained no improvement for visual defect after surgery. Preoperative hypopituitarism was detected in 11 cases (11/24, 45.8%), with 9 of them achieving clinical remission (81.8%) post-surgery and 2 remaining the same (18.2%). Headache was detected in 20 patients (20/24, 83.3%) on admission, and all of them relieved significantly after surgery (Supplementary Table 1). Gait unsteadiness or cognitive disturbance was found in 15 patients (15/24, 62.5%), and all of them improved to different extent post-surgery.

Intracapsular hemorrhage occurred in 2 cases (2/24, 8.3%), who underwent endoscopic transsphenoidal surgery and obtained subtotal resection (STR) of the tumors. They were both initially conscious, while one of them experienced craniotomy evacuation due to neurological deterioration. The other patient received conservative treatment with the hematoma resolved completely in 1 month. Postoperative intracranial infection was found in 1 case (1/24, 4.2%) after pterional surgery, who underwent intrathecal injection of antibiotics and recovered completely 1 month later. Cerebrospinal fluid (CSF) rhinorrhea was detected in 1 patient (1/24, 4.2%), which was resolved with conservative treatment. Four patients developed transient diabetes insipidus after surgery and recovered within 1 week. No death or symptoms related to cavernous sinus was noticed in our cases.

### Representative cases

### Case 1

**Figure 1:**
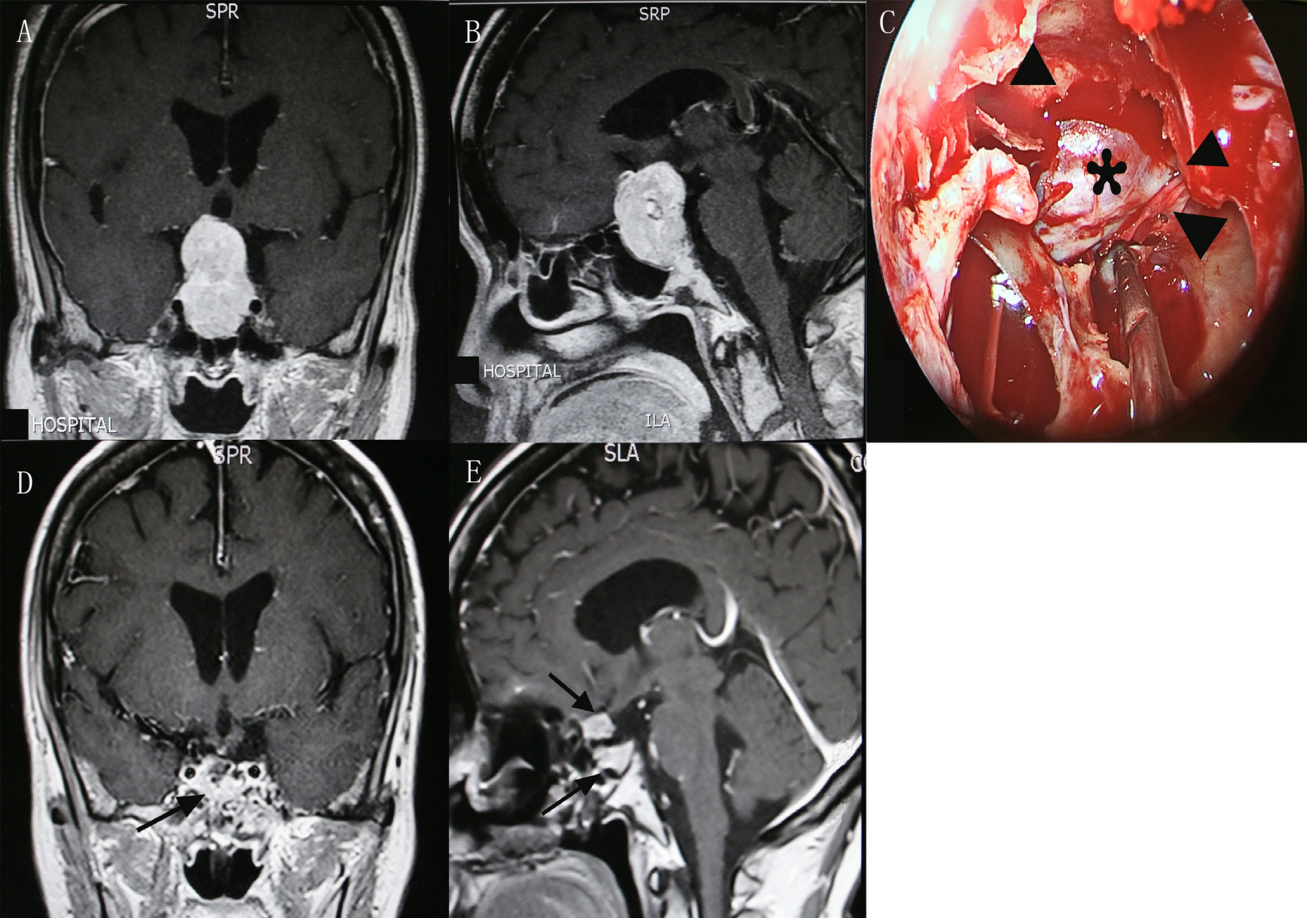
Contrast-enhanced MRI demonstrated preoperative coronal (**A**) and sagittal (**B**) views of a giant pituitary adenoma. The suprasellar adenoma (asterisk) was viewed under direct visualization after the opening of diaphragma sellae (triangle) from the endoscopy. (**C**) Postoperative coronal (**D**) and sagittal (**E**) views showed a small residual of tumor and fat graft (arrow) placed in the pituitary fossa. MRI, magnetic resonance imaging.

A 63-year-old female was admitted into our department with decreased visual acuity and bitemporal hemianopsia for 36 months. She also complained headache, impaired memory and unstable gait for 1 month. Preoperative magnetic resonance imaging (MRI) indicated a giant pituitary adenoma extending to suprasellar region and causing obstructive hydrocephalus (Figure 1A, 1B). Preoperative EVD was performed to relieve the hydrocephalus. Endoscopic transsphenoidal resection of the tumor was conducted under direct visualization (Figure 1C). Postoperative MRI confirmed a small residual of the tumor (Figure 1D, 1E). The postoperative course was uneventful. She obtained partial recovery of visual defect (preoperative/postoperative VIS: 77/34) and significant improvement of other symptoms at follow-up.

### Case 2

**Figure 2:**
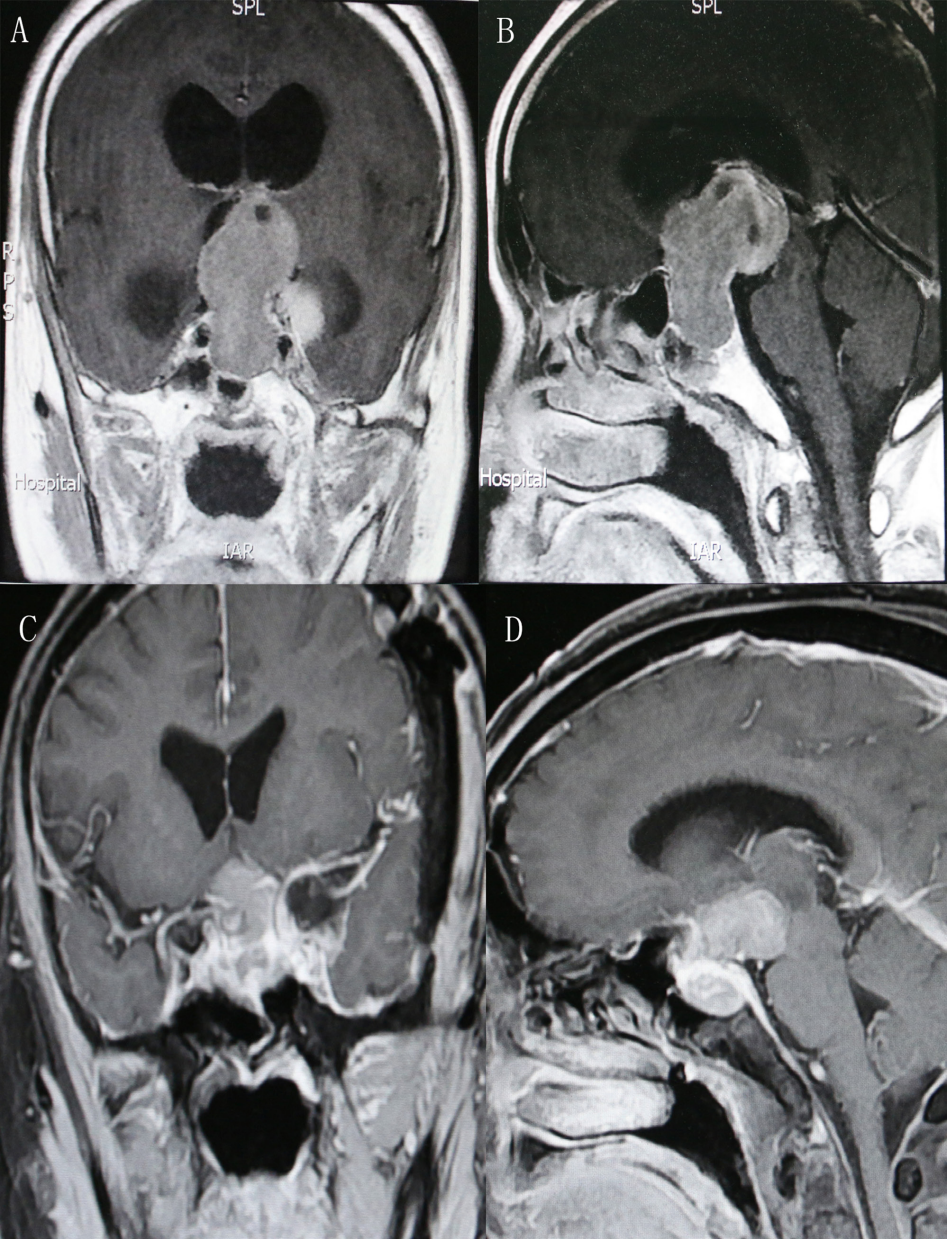
Preoperative coronal (**A**) and sagittal (**B**) contrast-enhanced MRI indicated a dumb-bell-shaped adenoma and obstructive hydrocephalus. Postoperative coronal (**C**) and sagittal (**D**) view demonstrated a STR of the tumor. MRI, magnetic resonance imaging; STR, subtotal resection.

This 56-year-old male was admitted with 15-month history of impaired visual acuity and bitemporal hemianopsia. Preoperative MRI suggested a huge dumb-bell-shaped pituitary adenoma and obstructive hydrocephalus (Figure 2A, 2B). He underwent a primary endoscopic transsphenoidal resection of the adenoma. The recurrence of tumor was confirmed 5 months later and a transcranial pterional surgery was adopted for him. Postsurgical VP shunt was conducted to deal with the sustained hydrocephalus. Contrast MRI indicated a STR of pituitary adenoma and resolution of hydrocephalus (Figure 2C, 2D). Notable improvement of his visual disturbance was achieved postoperatively.

## DISCUSSION

NFGPAs causing hydrocephalus is extremely rare with only 13 cases reported in previous literatures (Table 2) and it lacks of guidelines for management in the past years [9–12]. What’s more, conclusions from previous case reports remain inconsistent, especially on the timing of hydrocephalus management and surgical approaches of pituitary adenomas. Four of these adenomas were reported by Joshi et al, who applied transsphenoidal surgery for patients and considered preoperative EVD as necessary to ensure the safety of subsequent operation [9]. Verhelst et al reported 1 case of NFGPA with hydrocephalus, who underwent preoperative VP shunt and transcranial surgery with unsatisfactory resection. He received combined therapies of transsphenoidal surgery, radiation therapy and dopamine agonist cabergoline, and obtained significant vision improvement finally [11]. One case with a sudden loss of consciousness and a history of vision defect was treated by simultaneous transsphenoidal and transventricular endoscopic removal of pituitary adenoma. An EVD was placed intraoperatively. He finally obtained STR with relieved symptoms [10]. Nakao at al described 2 patients with NFGPAs causing hydrocephalus who were operated with endoscopic endonasal approach and recovered well after surgery. The management of hydrocephalus was not discussed in this study [12]. Baumann et al reported a patient with NFGPA extending to the interventricular foramen and causing hydrocephalus. She underwent endonasal transsphenoidal surgery and a VP shunt postsurgically for CSF rhinorrhea. Follow-up data suggested improved symptoms and normalization of visual fields [13]. Four patients were reported by Shenkin et al in year of 1973 with limited reference value due to its outdated diagnosis and treatment modalities [14].

**Table 2:** Summary of patients with NFGPAs in previous literatures

Study	Number. of cases	Sex/age	Symptoms on admission	Preoperative EVD or shunt	Surgery	Prognosis
Shenkin et al, 1973 [14]	4	F/50	Somnolence, right homonymous hemianopia	Torkildsen’s shunt	Right transfrontal craniotomy	Improved symptoms
		F/73	Altered personality, unsteadiness of gait	No	Ventriculoatrial shunt	Improved symptoms
		F/NK	Vision defect, headaches, partial bitemporal hemianopsia	Ventriculojugular shunt	Left transfrontal craniotomy	Died
		M/45	Altered personality, gait disturbance, bitemporal hemianopsia	Ventriculojugular shunt	Craniotomy	Free of symptoms
Verhelst J et al, 1998 [11]	1	M/61	Vision defect, somnolence	Ventriculoperitoneal shunt	Transcranial and transsphenoidal resection	Improved symptoms
Joshi et al, 2009 [9]	4	M/72	Vision defect, confusion	No	Transsphenoidal surgery	Death
		M/71	NK	EVD	Transsphenoidal surgery	Free of symptoms
		M/32	Headaches, confusion, vision defect	EVD	Transsphenoidal surgery	Vision defect
		M/42	Altered personality, vision defect	EVD	Transsphenoidal surgery	Vision defect
Nakao et al, 2010 [12]	2	M/58	Bitemporal hemianopsia, gait unsteadiness	No	Endoscopic endonasal transsphenoidal surgery	Improved symptoms
		NK/NK	Gait disturbance and cognitive dysfunction	NK	Endoscopic endonasal transsphenoidal surgery	Improved symptoms
Baumann et al, 2010 [13]	1	F/60	Vision defect, gait disturbance, urinary incontinence, memory deficits	No	Endonasal transsphenoidal surgery	Improved symptoms and normalization of visual fields
Koktekir et al, 2014 [10]	1	F/56	Headache, vision defect, sudden loss of consciousness	Intraoperative EVD	Simultaneous transsphenoidal and transventricular endoscopic surgery	Free of symptoms

Abbreviations: EVD, external ventricular drain; F, female; M, male; NFGPA, nonfunctioning pituitary adenomas; NK, not known.

In the present study, we evaluate the management of 24 patients with NFGPAs and hydrocephalus, and find that preoperative EVD could significantly reduce the need for postoperative shunt or EVD (*P* < 0.05). Therefore, we suggest preoperative placement of EVD according to clinical presentations to prevent neurological deterioration caused by hydrocephalus [11, 15]. Firstly, patients with giant pituitary adenomas are frequently admitted to emergent department due to symptoms of intracranial hypertension such as headache, vomiting, and even loss of consciousness. Preoperative EVD could significantly relieve these symptoms and secure subsequent surgery [11, 15]. Secondly, GTR could not be ensured whatever the surgical approaches are. The postoperative development of edema or intracapsular hematoma in the residual tumor might aggravate the obstructive hydrocephalus. Thirdly, brain edema owing to the release of vasopressin into CSF or postoperative displacement of residual tumor might prevent the remission of symptoms, recalling the need of postoperative shunt or EVD [11].

In our case series, GTR was achieved in 6 patients (33.3%) via endoscopic transsphenoidal approach and 4 (40%) via pterional approach, which was comparable to other reports [12, 16–18]. The main symptoms were headache, gait unsteadiness due to hydrocephalus, hypopituitarism and visual defect. In our report, 22 (91.7%) patients showed recovery of visual impairment, which was similar to previous studies [8, 12]. However, complete recovery was available in only 7 patients (29.2%), which might be related to the duration from visual impairment to surgery [12]. According to previous publications, the rate of postoperative complications ranged from 5% to 18% [16–19]. Two cases of intracapsular hematoma (8.3%), 1 intracranial infection (4.2%) and 1 CSF leakage (4.2%) were detected in our study.

Transsphenoidal approach is usually recommended for NFGPAs [2, 20, 21]. In particular, endoscopic transsphenoidal approach has the advantages of minimal invasion, operation under direct visualization, short anesthesia time and rapid recovery [10, 12]. Among patients undergoing transsphenoidal approach, GTR was achieved in 6 patients (33.3%), and vision improvement were available for 17 patients (94.4%). However, giant adenomas with extensive suprasellar invasion would present challenges for standard transsphenoidal approach due to the limited visualization of tumor outside the surgical corridor [12]. Several techniques including Valsalva maneuver, jugular compression are used to reduce intracranial hypertension and then induce the descent of suprasellar tumors into intrasellar region [16, 18, 22–25]. Although such techniques might assist in the resection of soft adenomas, their benefit for dumb-bell-shaped, fibrous tumors is limited. Other methods, such as extracapsular removal might have high risk of postoperative CSF leakage and injury to surrounding structures despite of possible assistance in the resection of some dumb-bell-shaped or fibrous adenomas [26–29].

For tumors that couldn’t be satisfactorily resected by transsphenoidal approaches, transcranial approach might be an alternative. Pterional surgery is applied to tumors extending to one side, making full use of the anterior area of optic chiasma, the space between optic nerve and internal carotid artery, and between internal carotid artery and oculomotor nerve. This approach could not only remove tumors but also avoid excessive traction of frontal lobe, as well as protect surrounding structures such as pituitary stalk, optic nerve and internal carotid artery [30, 31]. In the present case series, 4 cases (40%) achieved GTR via pterional approach, involving 2 patients of recurrent adenomas after transsphenoidal approach. Visual impairment recovered in 9 cases (90%).

Combined or two-stage surgery has been reported to be helpful in the removal of pituitary adenomas. Koktekir et al reported satisfactory resection of adenomas using simultaneous transsphenoidal and transventricular endoscopic approach [10]. Combined endoscopic transsphenoidal-transventricular approach was used in the resection of a giant pituitary macroadenoma, which might improve the GTR rate of adenomas [32]. As for two-stage surgery, the second surgery is usually conducted 2 or 3 months later, while disturbance of saddle areas and the use of stuffings might render the second surgery difficult. Four patients in our study suffered recurrent adenomas and underwent a secondary pterional surgery. Visual outcomes improved significantly for them. However, both combined and two-stage surgery would pose secondary injuries to patients. So we suggested alternative approaches only in case of recurrence or unsatisfactory first resection. Postoperative radiotherapy might help control the recurrence of adenomas, benefiting NFGPAs that could not be satisfactorily resected by one-stage surgery [2, 33].

To our acknowledgement, this case series is the largest case study regarding the management of NFGPAs complicated with hydrocephalus. However, because it is a retrospective study, there are several limitations. Firstly, the retrospective nature of this study brought unavoidable bias such as recall bias. Secondly, it is a long time span for our study. The diagnostic and therapeutic modalities vary significantly at the early and late phase of our study, which may affect the outcomes of patients. Lastly, we couldn’t draw more conclusions about the surgical approach, which is closely correlated with surgeons’ experience.

Conclusively, for patients of NFGPAs causing hydrocephalus, preoperative placement of EVD might avoid the need of a second shunt or EVD. Surgical approach should be decided based on the clinicoradiological features and surgeons’ experience for individualized treatment, and endoscopic transsphenoidal resection of adenomas was suggested for most NFGPAs. More large scale prospective and clinical studies were needed to confirm our findings and provide treatment options for patients with stratified needs.

## MATERIALS AND METHODS

### Patients population

We performed a retrospective study including 24 patients of NFGPAs complicated with hydrocephalus. All patients were admitted to Department of Neurosurgery, Changzheng Hospital, Second Military Medical University between January 2006 and December 2016. Clinical data, including demographic information, smoking, alcohol, tumor mass and extension, comorbidities (hypertension, diabetes, hyperlipidemia), clinical presentations, endocrine function, visual outcomes (visual acuity, visual field), enhanced MRI, preoperative or postoperative EVD/shunt, operators, surgical approach, outcome and complication were collected by 2 authors (D.F. Z., Z.X. L.) independently. The diagnosis of NFGPA with hydrocephalus was based on the clinical presentations and imaging findings. Informed consent was available for each patient. The present study was approved by the institutional ethics board of Changzheng Hospital.

### VIS

The mean duration from vision defect to surgery was 14.13 months (range: 1 to 36 months). We scored the preoperative and postoperative visual acuity and visual field separately for each patient according to the guidelines of the German Ophthalmological Society [12, 34]. The scores for both visual outcomes were then added to generate the VIS for all patients. To quantitively evaluate the recovery of vision defect after surgery, vision improvement was defined as preoperative VIS minus postoperative VIS.

### Treatment methods

Eighteen patients underwent endoscopic transsphenoidal resection of NFGPAs. Four of them experienced recurrence and a follow-up transcranial removal of pituitary adenomas. Ten patients received pterional surgery, including 4 cases with recurrence after transsphenoidal approach. For all patients, 7 of them were performed by operator 1 (K.W. H.), 10 by operator 2 (L.J. H.) and 7 by operator 3 (J.Y. W.). Preoperative EVD was performed for 9 patients, among whom 2 received pterional approach, 4 received transsphenoidal approach, 3 received both transsphenoidal approach and pterional approach. The extent of adenoma resection was defined as GTR or STR according to the postoperative MRI findings. Presence of any contrast-enhancing tumor on MRI suggested STR, while no evidence of tumor indicated GTR. The mean follow-up duration was 11.4 months (range: 2 to 32 months).

### Statistically analysis

Continuous or ordinal data were expressed as median and interquartile range. Binary data were reported as percentages and compared using fisher’s exact test. We used Wilcoxon signed rank test to compare paired or 2 independent groups and multiple independent groups were compared with Kruskal-Wallis test. Moreover, the Spearman correlation analysis was used to explore the relationship between tumor mass and vision improvement. *P* < 0.05 was considered as statistically significant. We performed all statistical analyses using SPSS (version 22.0; IBM, Inc., Chicago, IL, USA).

## SUPPLEMENTARY MATERIALS TABLE





## Abbreviations

CSF: cerebrospinal fluid
EVD: external ventricular drainage
GTR: gross total resection
MRI: magnetic resonance imaging
NFPAs: nonfunctioning pituitary adenomas
NFGPAs: nonfunctioning giant pituitary adenomas
STR: subtotal resection
VIS: visual impairment score
VP shunt: ventriculoperitoneal shunt.
