# Supplementary material for: Clinical features and management of nonfunctioning giant pituitary adenomas causing hydrocephalus
Source: Oncotarget. 2018 Jan 11;9(20):15409–17. doi: 10.18632/oncotarget.24171 (PMC5880614; doi:10.18632/oncotarget.24171)
Supplement: Supplementary file 1 [file oncotarget-09-15409-s001.pdf]

Clinical features and management of nonfunctioning giant pituitary adenomas causing hydrocephalus

SUPPLEMENTARY MATERIALS

Supplementary Table 1: Clinical features and management of 24 NFGPAs causing hydrocephalus.
See_Supplementary_Table 1
